# A novel radiation protection device based on tungsten functional paper for application in interventional radiology

**DOI:** 10.1002/acm2.12083

**Published:** 2017-04-19

**Authors:** Hajime Monzen, Mikoto Tamura, Kohei Shimomura, Yuichi Onishi, Shinichi Nakayama, Takahiro Fujimoto, Kenji Matsumoto, Kohei Hanaoka, Takeshi Kamomae

**Affiliations:** ^1^ Department of Medical Physics Graduate School of Medical Science Kindai University Osakasayama 589‐8511 Japan; ^2^ Clinical Radiology Service Division Kindai University Hospital Osakasayama 589‐8511 Japan; ^3^ Division of Clinical Radiology Service Okayama Central Hospital Okayama 700‐0017 Japan; ^4^ Clinical Radiology Service Division Kyoto University Hospital Kyoto 606‐8507 Japan; ^5^ Department of Therapeutic Radiology Nagoya University Graduate School of Medicine Nagoya 466‐8550 Japan

**Keywords:** fluoroscopy, interventional radiology, radiation protection, scattered radiation, tungsten functional paper

## Abstract

Tungsten functional paper (TFP), which contains 80% tungsten by weight, has radiation‐shielding properties. We investigated the use of TFP for the protection of operators during interventional or therapeutic angiography. The air kerma rate of scattered radiation from a simulated patient was measured, with and without TFP, using a water‐equivalent phantom and fixed C‐arm fluoroscopy. Measurements were taken at the level of the operator's eye, chest, waist, and knee, with a variable number of TFP sheets used for shielding. A Monte Carlo simulation was also utilized to analyze the dose rate delivered with and without the TFP shielding. In cine mode, when the number of TFP sheets was varied through 1, 2, 3, 5, and 10, the respective reduction in the air kerma rate relative to no TFP shielding was as follows: at eye level, 24.9%, 29.9%, 41.6%, 50.4%, and 56.2%; at chest level, 25.3%, 33.1%, 34.9%, 46.1%, and 44.3%; at waist level, 45.1%, 57.0%, 64.4%, 70.7%, and 75.2%; and at knee level, 2.1%, 2.2%, 2.1%, 2.1%, and 2.1%. In fluoroscopy mode, the respective reduction in the air kerma rate relative to no TFP shielding was as follows: at eye level, 24.8%, 30.3%, 34.8%, 51.1%, and 58.5%; at chest level, 25.8%, 33.4%, 35.5%, 45.2%, and 44.4%; at waist level, 44.6%, 56.8%, 64.7%, 71.7%, and 77.2%; and at knee level, 2.2%, 0.0%, 2.2%, 2.8%, and 2.5%. The TFP paper exhibited good radiation‐shielding properties against the scattered radiation encountered in clinical settings, and was shown to have potential application in decreasing the radiation exposure to the operator during interventional radiology.

## 1 Introduction

Lead has been employed in radiation protection in a clinical setting for many years; its uses include shielding for operator and patient protection in X‐ray radiography and computed tomography, as well as during electron beam radiotherapy. In addition, radiation‐shielding materials made of lead or lead equivalents are not flexible.^1^, ^2^ To overcome these problems, some researchers have explored ways of providing effective X‐ray protection using alternative materials.^3^, ^4^, ^5^, ^6^ In an attempt to reduce the weight of protection materials, several vendors have developed composite lead‐equivalent materials using mixtures of different elements such as lead, tin, copper, bismuth‐antimony, and yttrium.^7^, ^8^ Tungsten functional paper (TFP: Toppan Printing Co., Ltd., Tokyo, Japan), which is the paper with thickness of 0.3 mm and containing 80% tungsten powder by weight, has been developed as a lead free radiation shielding alternative, that has the advantages of paper in that it is easy to cut, fold, and stick onto other materials. These characteristics make it particularly flexible for many radiation‐protection applications, such as in radiation‐shielding surgical drapes, which have been reported based on bismuth and barium.^9^, ^10^, ^11^, ^12^, ^13^, ^14^, ^15^

In a previous study, we have shown that TFP has radiation‐shielding properties for various peak X‐ray kilovoltages in the range of 60–120 kVp.^16^ We found that 10 TFP sheets (3.0 mm) could have 0.48 ± 0.02 mm lead equivalent for 100 kVp x‐ray.^16^ Furthermore, in electron‐based radiotherapy, Fujimoto et al. reported an evaluation of the shielding characteristics of TFP against electron beams with clinically relevant energies and demonstrated that TFP is a useful radiation‐shielding material with potential clinical applications.^17^

Interventional radiology (IR) is a medical specialty that utilizes fluoroscopic guidance for the diagnosis and treatment of disease, and can involve the administration of relatively large doses of radiation. In IR, radiation exposure to the operator results mainly from scattered radiation from the patient.^18^, ^19^ Because the operator must stand close to patient during procedures and thus cannot utilize distance for radiation protection, occupational shielding is especially important. It is difficult for the operator to maintain a large distance of separation from the radiation source, because they must operate the equipment close to the patient. Moreover, during complex operations, a large number of x‐ray fluoroscopy images may be required. For these reasons, in IR, the operator is limited in terms of distance from the radiation source and the exposure time; therefore, shielding is especially important.

In IR, both the patient and operator face risks from radiation exposure. Patients are primarily at risk of potential skin effects from prolonged radiation exposure. Operators and staffs are at risk of radiation‐induced cataracts and potentially stochastic long‐term cancer risk.^20^ These risks are minimized in appropriately controlled conditions; however, radiation hazards for workers conducting IR in inappropriate controlled conditions have been reported.^20^ One principle of working with radiation is to ensure that the radiation exposure is “as low as reasonably achievable”.^21^ In order to achieve this, various practical protective measures have been developed to reduce the backscatter radiation from the patient, including ceiling mounted shields and table mounted leaded drapes.^22^ Chida et al. measured operator air kerma rates using a luminescence dosimeter (Luxel Badge; Landauer Inc., Glenwood, IL, USA) during cardiac catheterization procedures, and found that the use of both the table and the ceiling lead drape reduced the occupational radiation exposure by approximately 42%.^23^

The purpose of the present study was to investigate and measure the utility of a novel TFP as a new method to reduce the operator's radiation exposure from scattered X‐ray radiation arising from the patient during IR procedures. We measured the reduction in the air kerma rate for the operator using various thicknesses of TFP, and compared measurements and computed simulations using the particle and heavy‐ion transport code system (PHITS) 24 Monte Carlo (MC) simulation. The physical measurement of the air kerma rate from a phantom was difficult; this was because the air kerma rates for scattered radiation from the phantom were very low.^25^

## 2 Materials and methods

**Figure 1:**
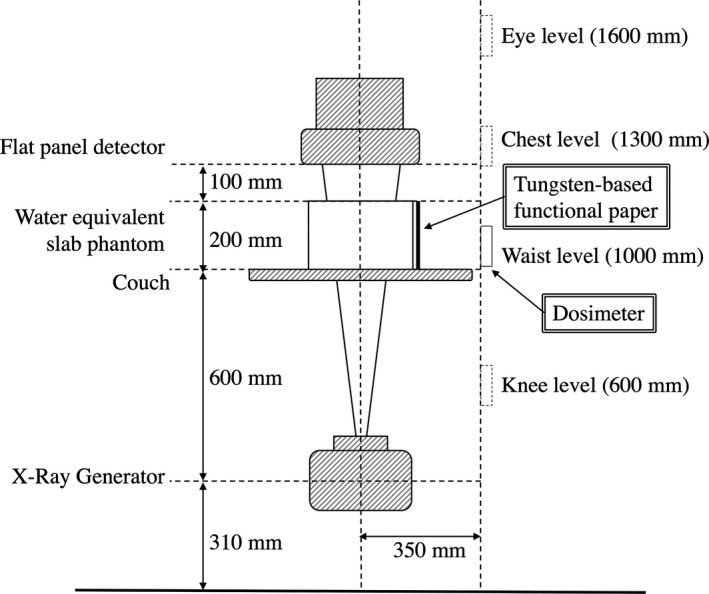
A schematic diagram of the measurement geometry for scattered radiation from the water equivalent slab phantom. The TFP was taped to the operator side of the phantom and the number of TFP sheets was varied through 1, 2, 3, 5, and 10.

The TFP was constructed from tungsten powder on cellulose‐based paper, with a density of 0.06–0.21 g/cm^2^ and a thickness of 0.3 mm per sheet. This material has similar characteristics to paper, and can be cut, folded, and glued, so that it can take various forms. Figure 1 shows a schematic diagram of the measurement geometry. A C‐arm digital angiography system (AXIOM‐ArtisdFC: Medical Solutions, Erlangen, Germany) was used with the tube located beneath the table holding water equivalent slabs to simulate the patient. The X‐ray equipment has two modes: fluoroscopy mode and image acquisition (or cine) mode. The X‐ray tube has an added filtration of a 2.5 mm of Al and 0.6 mm of Cu and a 900‐mm separation between the source and the detector. The patient table was in the beam path, and was modeled as 0.86 mm Aluminum equivalent for the Monte Carlo simulations. The field of view was 180 × 180 mm^2^, and the detector entrance dose rates were 32 nGy/pulse in fluoroscopy mode (with 15 frames s^−1^ and a pulse width of 3.6 ms) and 0.2 μGy/frame in cine mode (with 15 frames s^−1^ and a pulse width of 6.2 ms), which were measured using an area detector of an ionization chamber (Diamentor K2S; PTW, Freiburg, Germany) during IR automatically. Table 1 lists the imaging parameters used in this study.

**Table 1:** Imaging parameters for the C‐arm digital angiography system (AXIOM‐ArtisdFC: Medical Solutions, Erlangen, Germany)

Mode	Fluoroscopy	Cine
Pulse/frame rate (s^−1^)	15	15
Pulse width (s)	3.6 × 10^‐3^	6.2 × 10^‐3^
Tube voltage (kV)	100	66
Tube current (mA)	144	333
ED (mGy pulse^−1^ or mGy frame^−1^)	32 × 10^‐6^	0.2 × 10^‐3^
DDO (%)	50	50

ED, detector entrance dose; DDO, dynamic density optimization.

This is a post‐processing parameter that functions in real‐time by harmonizing the distribution of gray steps in the image.

Four water equivalent slabs (TM phantom: Taisei Medical Inc. Kyoto, Japan) were placed on the table to simulate a typical patient. Each slab had dimensions of 300 mm (length) × 300 mm (width) × 50 mm (height), for a total phantom thickness of 200 mm. The survey meter of a solid state detector (Unfors Xi: Unfors Billdal, Sweden) shaped like a lollipop was used to measure the scattered radiation. This device is useful to measure the low level radiation such as scattered radiation from x‐ray tubes or examination rooms. We measured the air kerma rates and the locations of the measurements were at heights corresponding to the eyes (1600 mm from the floor), chest (1300 mm), waist (1000 mm) and knee (600 mm) of the operator; these values were based on Japanese male average height. The statistical errors were standard deviations estimated from five times measurements at each height. The TFP was cut into 300 × 200 mm^2^ strips and taped to the operator side of the TM phantom, and scattered radiation was measured with and without the TFP. The number of TFP sheets were varied through 1, 2, 3, 5, and 10, and the measurements were repeated twice. Data regression was performed using the Igor Pro (Version 6.33J: WaveMetrics, Lake Oswego, OR, USA) software package, and measurements were compared with the MC simulations because it was difficult to measure the air kerma rate for the operator during angiography, the air kerma rate for scattered radiation from the phantom were very low. We used the particle and heavy‐ion transport code system (PHITS; version 2.64: Japan Atomic Energy Agency, Japan), which has been used in many fields related to particle and heavy ion transport, including accelerator technology, radiotherapy, space radiation, and radiation protection.^19^ The geometry as shown in Fig. 1 was modeled with PHITS, with the patient table modeled as aluminum of a 0.86 mm thickness. The X‐ray spectrum was calculated using the Birch formula ^(28)^, with cut‐off kinetic energies of 1 keV for photons and 66 keV for electrons. The measured dose distribution at each height was simulated over a volume of 20.0 (length) × 10.0 (width) × 10.0 (height) mm^3^. The simulation domain was discretized into 180 points in the vertical direction (giving a grid size of 1.0 cm from the floor to a height of 180.0 cm). The number of layers of TFP was varied through 0, 1, 2, 3, 5, and 10. The statistical uncertainties in the PHITS calculations were within 3% at waist level.

## 3 Results

Table 2 lists the results of measurements involving 0, 1, 2, 3, 5, and 10 sheets of TFP. The measured air kerma rates were decreased when an increasing number of TFP sheets were used for the eye, chest, and waist protection. However, at the knee level the measured dose varied little as a function of the number of TFP sheets. The trends were similar using fluoroscopy mode and cine mode.

**Table 2:** Measured air kerma rates using different numbers of TFP sheets

Mode	Fluoroscopy (*μ*Gy/min)						Cine (*μ*Gy/min)					
Number of TFP sheets	0	1	2	3	5	10	0	1	2	3	5	10
Eye level	4.5	3.4	3.1	2.9	2.2	1.9	30.1	22.6	21.1	17.5	14.9	13.2
Chest level	14.7	10.9	9.8	9.5	8.0	8.2	98.5	73.6	65.9	64.1	53.2	54.9
Waist level	33.4	18.5	14.4	11.8	9.5	7.6	235.4	137.7	101.2	83.9	68.9	58.4
Knee level	36.3	35.5	36.8	35.5	35.3	35.4	240.4	235.4	235.1	235.4	235.4	235.3

**Figure 2:**
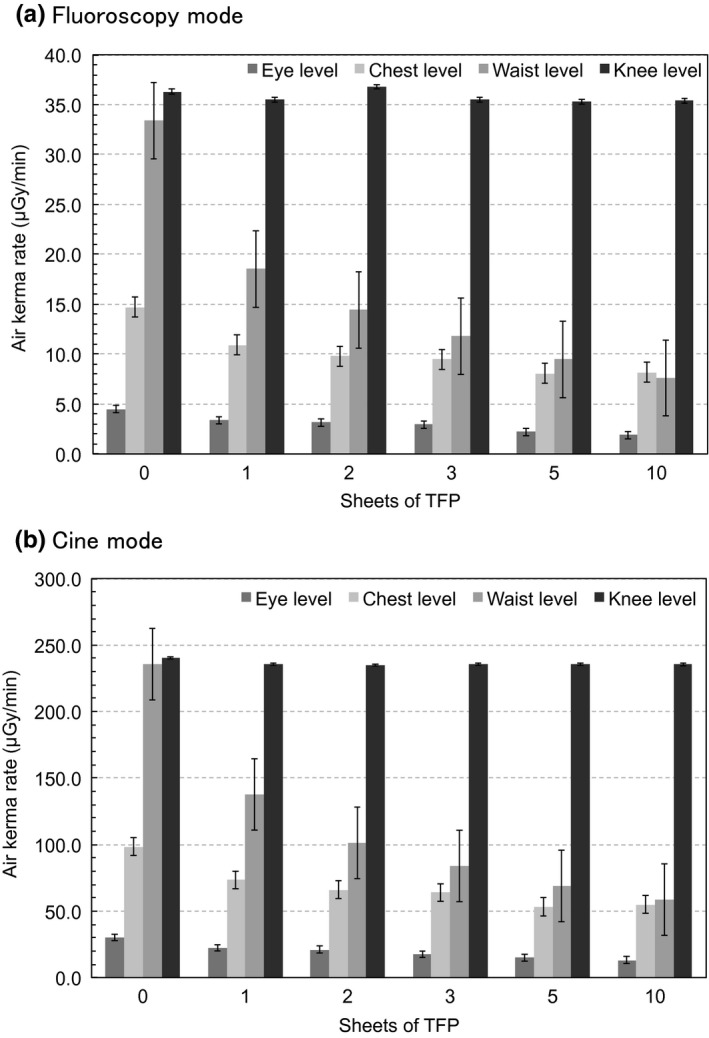
The air kerma rates as a function of the number of TFP sheets at eye‐, chest‐, waist‐, and knee‐level measurements.

**Figure 3:**
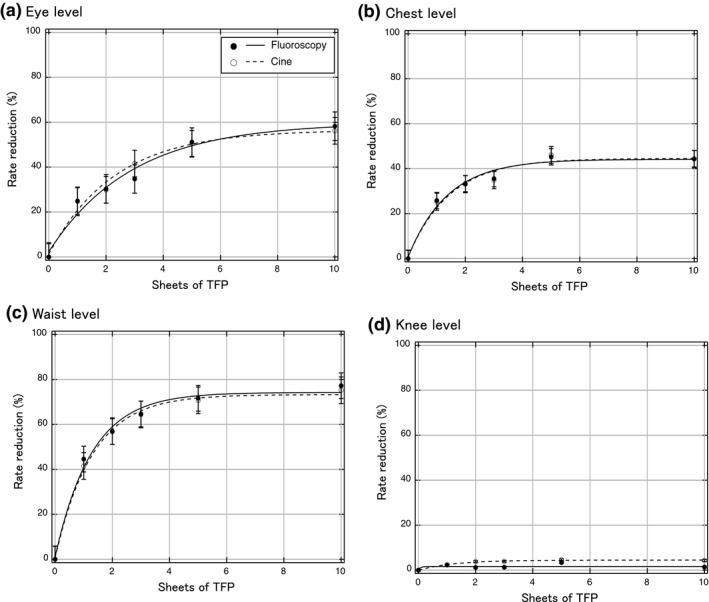
The measured percentage reduction in the radiation dose as a function of the number of layers of TFP, with curve fitting. (a) At eye level, (b) at chest level, (c) at waist level, and (d) at knee level.

**Figure 4:**
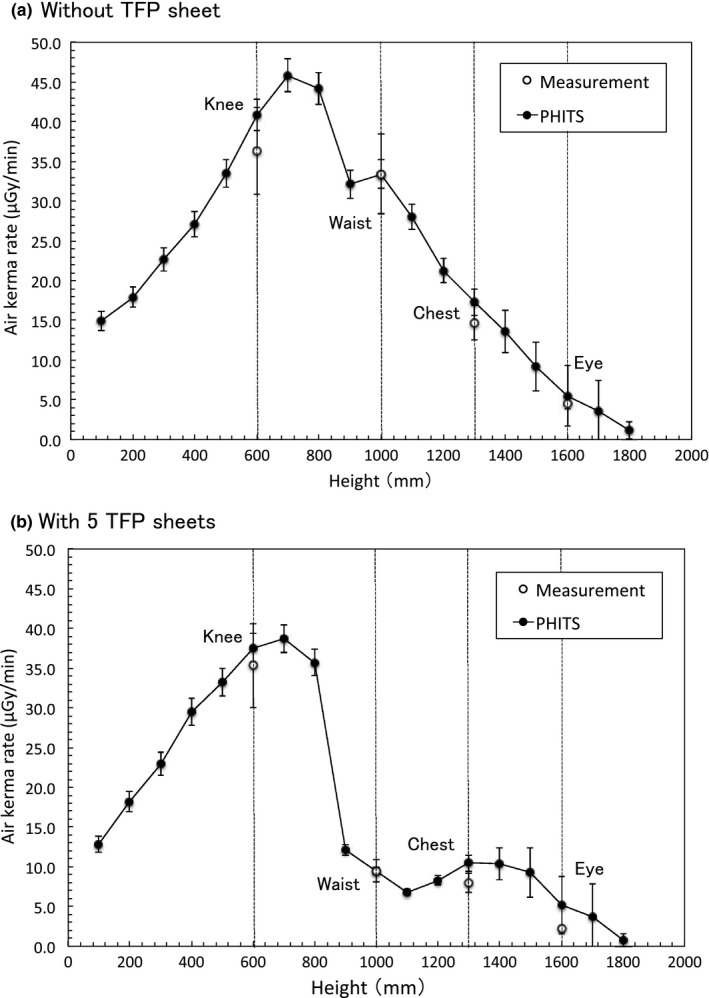
The relative air kerma rate distributions normalized to the waist level values as a function of height, obtained from measurements and PHITS simulations. (a) Without TFP and (b) with five TFP sheets.

Figure 2 shows the air kerma rate as a function of the number of TFP sheets at eye, chest, waist, and knee level. Figure 3 shows the fitted curves for the percent reduction in rate at each level calculated from Fig. 2 data using Igor Pro software. With five sheets of TFP the rate reduction in the air kerma rates relative to those measured with no TFP shielding using cine mode were 50.4%, 46.1%, 70.7%, and 2.1% at eye, chest, waist, and knee levels, respectively. Using fluoroscopy mode, the respective rate reduction in the air kerma rates as compared with no TFP shielding were 51.1%, 45.2%, 71.7%, and 2.8% at eye, chest, waist, and knee levels, respectively. Figure 4(a) shows a comparison between the calculations using PHITS and the measured data for the relative air kerma distributions at operator height without TFP shielding, and Fig. 4(b) shows the relative air kerma distributions using five sheets of TFP. The relative air kerma distributions at operator height were normalized to the value at waist level for each distribution. The MC simulations were in close agreement with our measurements, which the differences for each average were within 3% (Fig. 4).

## 4 Discussion

We evaluated the radiation‐shielding ability of TFP regarding scattered radiation from a TM phantom. Although the statistical uncertainties of our measurements were approximately 15%, their accuracy was verified by comparing them with MC simulations because the air kerma rate measurements are difficult for all locations and thicknesses of TFP as shown Fig. 4. TFP was effective in shielding the operator from scattered radiation during IR. Using only one TFP sheet it was possible to reduce scattered radiation from the TM phantom by > 40%. When the number of TFP sheets was increased from 5 (1.5 mm) to 10 (3.0 mm), a further reduction of only 10% was measured, indicating that five TFP sheets exhibited saturated shielding ability concerning the lateral scattered radiation from the TM phantom. The reduction in the dose rate at chest and eye level was smaller than that at waist level, because the direct scatter attenuated by the TFP was less due to scatter angle. In addition, at knee level, the air kerma rate could not be decreased by the TFP sheets placed at the side of the phantom body, because the scattered radiation from direct irradiation did not pass the TFP sheets.

Further development of TFP for applications such as surgical drapes will require additional modifications, including single‐sided water or blood absorption layers, and a waterproofing layer on the rear. We have already attempted to create waterproof layers on TFP sheets. The simplest method is to laminate water‐repellent material to one or both sides of the TFP sheet. TFP has the potential to be a practical substitute for existing protective devices such as lead drapes for the treatment table and ceiling, surgical drapes, and to serve as a general‐purpose radiation‐shielding material.

## 5 Conclusion

We have described measurements and simulations of the radiation‐shielding ability of TFP for IR, especially concerning scattered radiation. We expect that TFP will play an important role in reducing the level of scattered radiation more flexibly than lead, because TFP is easy to cut, paste, and roll as a result of its paper‐like properties.

## conflict of Interest

Hajime Monzen has a consultancy agreement with, and financial interest in, TOPPAN PRINTING CO., LTD, Tokyo.

## References

[1] Rempel D . The lead‐exposed worker. JAMA. 1989;262:532–534. 2661879

[2] Needleman H . Lead poisoning. Annu Rev Med. 2004;55:209–222. 14746518 10.1146/annurev.med.55.091902.103653

[3] Hubbell JH , Seltzer SM . Tables of X‐ray mass attenuation coefficients and mass energy‐absorption coefficients 1 keV to 20 MeV for elements Z=1 to 92 and 48 additional substances of dosimetric interest. NISTIR 5632, National Institute of Standards and Technology; 1995.

[4] Murphy PH , Wu Y , Glaze SA . Attenuation properties of lead composite aprons. Radiology. 1993;186:269–272. 8416577 10.1148/radiology.186.1.8416577

[5] McCaffrey JP , Shen H , Downton B , Mainegra‐Hing E . Radiation attenuation by lead and nonlead materials used in radiation shielding garments. Med Phys. 2007;34:530–537. 17388170 10.1118/1.2426404

[6] McCaffrey JP , Mainegra‐Hing E , Shen H . Optimizing non‐Pb radiation shielding materials using bilayers. Med Phys. 2009;36:5586–5594. 20095271 10.1118/1.3260839

[7] Christodoulou EG , Goodsitt MM , Larson SC , et al. Evaluation of the transmitted exposure through lead equivalent aprons used in a radiology department, including the contribution from backscatter. Med Phys. 2003;30:1033–1038. 12852526 10.1118/1.1573207

[8] Chatterson LC , Leswick DA , Fladeland DA , Hunt MM , Webster ST . Lead versus bismuth‐antimony shield for fetal dose reduction at different gestational ages at CT pulmonary angiography. Radiology. 2011;260:560–567. 21555348 10.1148/radiol.11101575

[9] Iqtidar AF , Jeon C , Rothman R , Snead R , Pyne CT . Reduction in operator radiation exposure during transradial catheterization and intervention using a simple lead drape. Am Heart J. 2013;165:293–298. 23453095 10.1016/j.ahj.2012.10.002

[10] Ertel A , Nadelson J , Shroff AR , Sweis R , Ferrera D , Vidovich MI . Radiation dose reduction during radial cardiac catheterization: evaluation of a dedicated radial angiography absorption shielding drape. ISRN Cardiol. 2012;2012:769167. 22988525 10.5402/2012/769167 PMC3439952

[11] Politi L , Biondi‐Zoccai G , Nocetti L , et al. Reduction of scatter radiation during transradial percutaneous coronary angiography: a randomized trial using a lead‐free radiation shield. Catheter Cardiovasc Interv. 2012;79:97–102. 21520391 10.1002/ccd.22947

[12] Murphy JC , Darragh K , Walsh SJ , Hanratty CG . Efficacy of the RADPAD protective drape during real world complex percutaneous coronary intervention procedures. Am J Cardiol. 2011;108:1408–1410. 21861961 10.1016/j.amjcard.2011.06.061

[13] Brambilla M , Occhetta E , Ronconi M , Plebani L , Carriero A , Marino P . Reducing operator radiation exposure during cardiac resynchronization therapy. Europace. 2010;12:1769–1773. 21097481 10.1093/europace/euq356

[14] Simons GR , Orrison WW . Use of a sterile, disposable, radiation‐absorbing shield reduces occupational exposure to scatter radiation during pectoral device implantation. Pacing Clin Electrophysiol. 2004;27:726–729. 15189526 10.1111/j.1540-8159.2004.00520.x

[15] Germano JJ , Day G , Gregorious G , Natarajan V , Cohen T . A novel radiation protection drape reduces radiation exposure during fluoroscopy guided electrophysiology procedures. J Invasive Cardiol. 2005;17:469–472. 16145234

[16] Monzen H , Tamura M , Hanaoka K , Matsumoto K , Hayakawa M . Development and application of radiation shielding paper. Ionaizing Radiat. 2016;41:139–143.

[17] Fujimoto T , Monzen H , Nakata M , et al. Dosimetric shield evaluation with tungsten sheet in 4, 6, and 9 MeV electron beams. Phys Med. 2014;30:838–842. 24953537 10.1016/j.ejmp.2014.05.009

[18] Schueler BA . Operator shielding: how and why. Tech Vasc Interv Radiol. 2010;13:167–171. 20723831 10.1053/j.tvir.2010.03.005

[19] Ito H , Hosoya T , Eguchi Y , Adachi M , Watanabe Y , Yamaguchi K . Analysis of radiation scatter during angiographic procedures: evaluation of a phantom model and a modified radiation protection system. J Vasc Interv Radiol. 1999;10:1343–1350. 10584649 10.1016/s1051-0443(99)70241-1

[20] Mettler FA , Koenig TR , Wagner LK , Kelsey CA . Radiation injuries after fluoroscopic procedures. Semin Ultrasound CT MR. 2002;23:428–442. 12509113 10.1016/s0887-2171(02)90014-4

[21] NCRP Implementation of the principle of as low as reasonably achievable (ALARA) for medical and dental personnel. NCRP Report 107; 1990.

[22] Durán A , Hian SK , Miller DL , Heron JL , Padovani R , Vano E . Recommendations for occupational radiation protection in interventional cardiology. Catheter Cardiovasc Interv. 2013;82:29–42. 23475846 10.1002/ccd.24694

[23] Chida K , Morishima Y , Katahira Y , Chiba H , Zuguchi M . Evaluation of additional lead shielding in protecting the physician from radiation during cardiac interventional procedures. Nihon Hoshasen Gijutsu Gakkai Zasshi. 2005;61:1632–1637. 16395238 10.6009/jjrt.kj00004022974

[24] Sato T , Niita K , Matsuda N , et al. Particle and heavy ion transport code system PHITS, version 2.52. J Nucl Sci Technol. 2013;50:913–923.

[25] Siiskonen T , Tapiovaara M , Kosunen A , Lehtinen M , Vartiainen E . Monte Carlo simulations of occupational radiation doses in interventional radiology. Br J Radiol. 2007;80:460–468. 17151067 10.1259/bjr/26692771

[26] Ministry of Health, Labor and Welfare The national health and nutrition survey in Japan; 2012.

[27] Birch R , Marshall M . Computation of bremsstrahlung x‐ray spectra and comparison with spectra measured with a Ge(Li) detector. Phys Med Biol. 1979;24:505–517. 461510 10.1088/0031-9155/24/3/002

